# Benign Palatine Tonsil Volume Variation Following Bilateral Tonsillectomy in Adults

**DOI:** 10.7759/cureus.7288

**Published:** 2020-03-16

**Authors:** Alexandra States, Simon Kirby

**Affiliations:** 1 Pathology, Eastern Health/Memorial University of Newfoundland, St. John's, CAN

**Keywords:** tonsillectomy, benign tonsils, tonsillar asymmetry, tonsils, bilateral tonsillectomy, adult tonsillectomy, adult tonsils

## Abstract

Background

There is a lack of data on normal size discrepancy in benign tonsils. The current school of thought for otolaryngologists is to remove tonsils that look clinically asymmetric on the basis of occult malignancy. However, many of these tonsils turn out to be benign after microscopic evaluation. The data in this article provide a reference range of size variation that can be seen in benign adult tonsils. Such new information can be incorporated into the surgeon’s preoperative discussion with patients with respect to informed consent and patient reassurance.

Methods

A chart review was conducted to identify pathology-proven benign bilateral tonsillectomies in the adult population. The review timeframe was from January 2012 to December 2017 (inclusive). All patients underwent surgery in an Eastern Health facility in Newfoundland and Labrador, Canada. In total, 403 cases were identified that fulfilled the inclusion criteria.

Results

Out of the 403 cases studied, the average tonsillar volume was 42.81 cm^3^. When differentiating between men and women, it can be seen that men have a higher average tonsil size (52.4 cm^3^) than women (37.85 cm^3^). The average difference in tonsil volume for all cases was 24.3%, with a standard deviation of 19.2%. Moreover, for men, the average difference in tonsil volume was 24.2%, with a standard deviation of 19.74%. Similarly, for women, the average difference in tonsil volume was 24.36%, with a standard deviation of 18.94%.

Conclusions

Findings from this study show that, on average, benign tonsils can vary in size by approximately 24% and that such a difference does not necessarily indicate malignancy.

## Introduction

Background

Informed consent is a foundational element of patient-centered care. This becomes especially relevant during the decision-making process for surgery. There is no doubt that today’s patient is more informed than ever, and they will ask physicians to corroborate their online research or rely on them solely for information. For the ENT (ear, nose, and throat) surgeon having a conversation regarding asymmetric tonsils and the risk of malignancy, there are little data available. Specifically, there is scant information about size discrepancy in benign tonsils. Our study aims to fill this knowledge gap and provides clinicians with relevant information that can be shared during the informed consent process for tonsillectomy.

Tonsils are collections of lymphoid tissue located at the back of the throat. After childhood, their role in the immune system is negligible. By far, the majority of tonsillectomies occur in children; however, adults also undergo this procedure. The two most common reasons for an adult to have their tonsils removed are obstructive sleep apnea and recurrent tonsillitis [1]. While tonsillectomies are typically seen as a relatively low-risk surgical procedure, with bleeding and pain being the two most commonly encountered perioperative issues, potentially life-threatening postoperative bleeding has been reported in 1.5-5% of patients [2]. Therefore, the decision to undergo a tonsillectomy should be taken seriously.

Asymmetric palatine tonsils are frequently removed due to clinical suspicion for malignancy. Following a tonsillectomy, specimens are grossed and examined microscopically. During grossing, tonsils are measured, and the presence of malignancy is determined through microscopy. This process most often reveals benign symmetric or asymmetric tonsils. In fact, studies show that the clinical examination finding of unilateral tonsillar enlargement does not necessarily indicate malignancy [3-4]. For example, Cinar discovered that oftentimes the smaller tonsil was actually the one that contained the malignant cells [3]. Building on this work, Sunkaraneni et al. concluded that unilateral tonsillar enlargement without other clinical symptoms did not necessarily indicate the presence of malignancy [5]. Syms et al. reported that at their institution, only 0.35% of patients with unilateral tonsillar enlargement had occult malignancy. In addition, they found that the clinically estimated size and grossly measured volume had a concordance rate of 60.5%, highlighting the inaccuracies with the physical assessment [6].

Further literature review reveals several studies that evaluate the presence of malignancy in asymmetric tonsils but none that calculate the variation in size of benign tonsils [1,6-7]. Therefore, more research is needed to establish a baseline of what variation in tonsil size can be expected in benign tonsils. This information can then be incorporated into the informed consent discussion.

Purpose

The purpose of this study is to calculate the variation in tonsil volume that exists between two benign tonsils in the same patient. Findings from this study may provide clinicians with information regarding normal volume variation in benign tonsils. These data then can be used during the informed consent discussion with patients. Moreover, the vast majority of research regarding tonsillectomies is conducted in the pediatric population [8]. Therefore, this project would add to the scant body of research in adult tonsillectomies and provide clinicians with much-needed data they can cite during the consent process.

## Materials and methods

Data Collection

The first step to this project was to conduct a chart review to identify pathology-proven benign bilateral tonsillectomies in the adult population. An extensive computer search of anatomical pathology reports was conducted using key words and phrases such as “benign”, “no evidence of malignancy”, “reactive”, and “hyperplasia”. While microscopic evaluations of the integrity of the tonsils were not possible, any tonsils with piecemeal excision, those found to be positive for malignancy and/or lymphoma, and any reports with typographical or dictational errors in relation to measurements were excluded from this study. An adult patient was defined as an individual aged 18 to 70 years at the time of surgery. The review timeframe was from January 2012 to December 2017 (inclusive). The start date was selected due to a system-wide change in reporting that was implemented at the start of 2012. The stop date was selected based on the time of data collection, which was February 2018. All patients underwent surgery in an Eastern Health facility in Newfoundland and Labrador, Canada. In total, 403 cases were identified that fulfilled the inclusion criteria. Data extracted from these cases included patient age, sex, tonsil dimensions at the time of grossing, and pathological diagnosis. Informed consent was not obtained, as this study was a retrospective review of secondary data. The Newfoundland and Labrador Health Research Ethics Board granted full ethics approval for this project.

Tonsil Analysis

After obtaining the data set, the next step was to determine tonsil volume and variance. Tonsillar volume was calculated using the validated ellipsoid method as per the equation below [9]:

$V = (4/3)*\pi*abc$

In the preceding equation, a, b, and c are the ellipsoidal height, width, and depth of the tonsil, respectively.

Given that the tonsillar volume was a critical parameter in the study, it is important to consider potential organ shrinkage during conservation and storage. Tonsils were stored in 10% neutral buffered formalin. While shrinkage is possible, both tonsils were equally exposed to the environment. As such, shrinkage would be expected to be proportionate to the original volume, and a qualitative comparison of the relative volume was deemed suitable.

Variation between tonsils was then calculated as an average percent difference between the two tonsils. This percent difference was then averaged for all samples and for the male and female groups.

## Results

Of the 403 cases studied, the average tonsillar volume was 42.81 cm^3^ (Table 1). However, when differentiating between men and women, it can be seen that men have a higher average tonsil size (52.4 cm^3^) than women (37.85 cm^3^).

**Table 1:** Average tonsil size and difference in volume for cases studies

Group	Average Tonsil Size (cm^3^)	Average Difference in Tonsil Volume	Standard Deviation	Number of Samples
All	42.81	24.30%	19.20%	403
Men	52.4	24.20%	19.74%	141
Women	37.65	24.36%	18.94%	262

**Figure 1:**
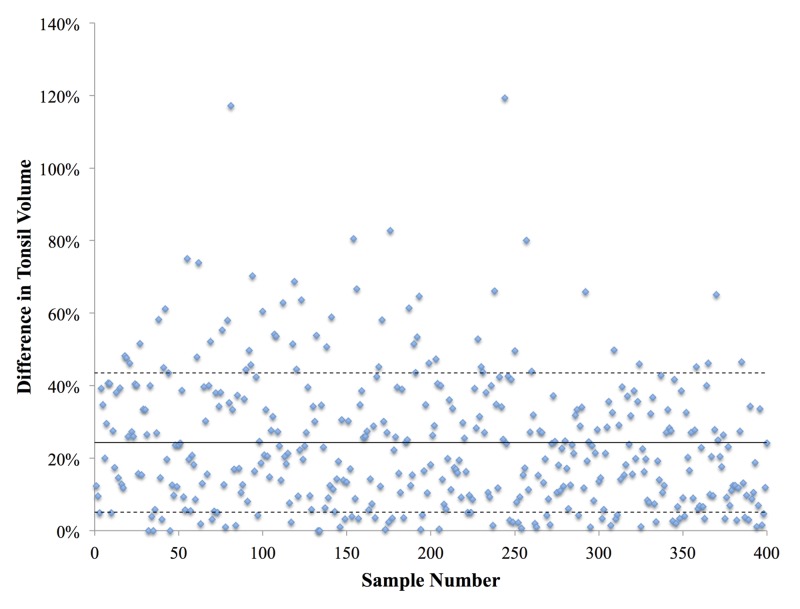
Percent difference in tonsil volume for the 403 samples considered The solid line represents the average tonsil volume, and the dotted lines represent ±1 standard deviation

**Figure 2:**
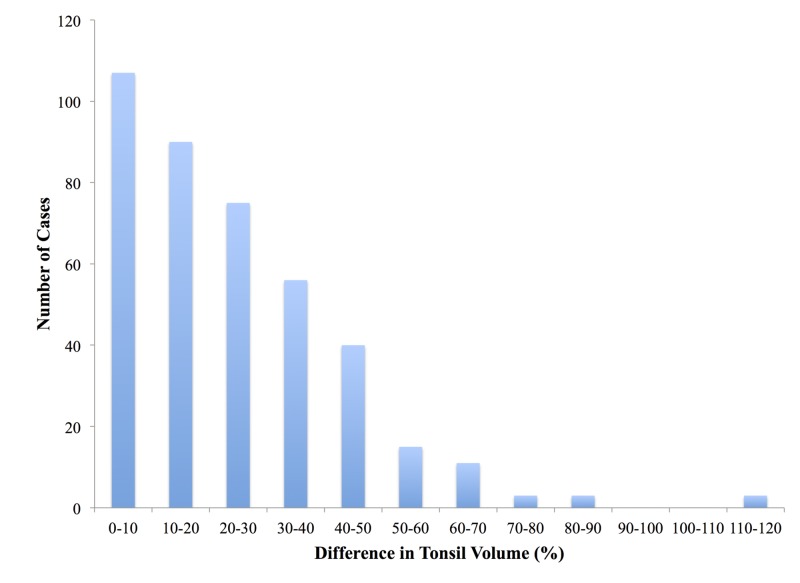
Number of cases within a specified percent difference in tonsil volume range

The average difference in tonsil volume for all cases was 24.3%, with a standard deviation of 19.2% (Table 1, Figure 1). Moreover, for men, the average difference in tonsil volume was 24.2%, with a standard deviation of 19.74%. Similarly, for women, the average difference in tonsil volume was 24.36%, with a standard deviation of 18.94%. Furthermore, of the 403 cases evaluated, 272 had a difference in tonsil volume between 0% and 30%, with only 35 cases having a difference in tonsil volume above 50% (Figure 2).

## Discussion

Overall, based on the 403 cases analyzed, this study found that benign tonsils can have a significant variation in size in the same patient (standard deviation of 20%). Moreover, even though male tonsils clearly had a larger average tonsil volume than female tonsils, their average volume difference was nearly identical (19.7% and 18.9%, respectively). Therefore, standard variation in tonsils is consistent for both male and female patients. Of the 403 samples, 286 were within 1 standard deviation of each other (±19.4%). Therefore, tonsils with a volume variation of less than 43.5% are unlikely to be malignant. However, 57 samples were above 1 standard deviation, 3 of which had a volume difference of over 100%. As a result, large differences cannot automatically be assumed to be malignant.

Findings from this study can also influence standards of care in clinical practice. For example, the standard of care dictates that an observable discrepancy in size on physical examination (such as 20%) warrants assessment and most likely results in excision. The results indicate that tonsillar volume variation does not always denote a sinister diagnosis. This information should be included in an informed consent discussion with the patient. That being said, it is unclear what these results might mean to clinicians with respect to management. Perhaps, for now, this finding is purely of academic interest but provides a foundation for which future studies can build upon.

Furthermore, this study did not look at the reason for the tonsillectomy. Despite having a benign tissue diagnosis, such as follicular hyperplasia, the patient may have been suffering from recurrent tonsillitis and he or she would have had the surgery electively. A future studying deciphering the relationship between the indication for surgery and pathological diagnosis would be prudent. In particular, a study focusing on asymmetry as a contributing deciding factor for surgery would be especially interesting. Another potential limitation in this study is the population sample considered. All participants underwent surgery in an Eastern Health hospital in Newfoundland and Labrador. Historically, the patient population in this region is relatively homogenous; therefore, the translatability of the results to other populations may be unreliable. Finally, as discussed above, the methods used to remove and store the tonsils may have potentially contributed to shrinkage. However, given that both tonsils were removed and stored using equivalent methods, the relative percent difference remains a valuable consideration. To allow for relevant quantitative results, future work can consider the effect of removal and storage method on tonsil volume.

## Conclusions

In conclusion, this study used a detailed chart review to identify more than 400 cases of bilateral tonsillectomies of benign tonsils. Findings from this study show that significant variations exist between benign tonsils and that this difference should not necessarily be a cause for concern. These results provide new, clinically relevant information that physicians can share with patients during the informed consent process. Understandably, the risk of malignancy is frightening to the majority of patients and contributes to the decision to proceed with surgery. In an effort to improve the psychological well-being of these patients, our data can be cited as a reassurance that asymmetric tonsils are a common finding and that this asymmetry is likely benign.
